# Concomitant Hemophagocytic Lymphohistiocytosis and Cytomegalovirus Disease: A Case Based Systemic Review

**DOI:** 10.3389/fmed.2022.819465

**Published:** 2022-04-19

**Authors:** Linn Åsholt Rolsdorph, Knut Anders Mosevoll, Lars Helgeland, Håkon Reikvam

**Affiliations:** ^1^Department of Clinical Science, Faculty of Medicine, University of Bergen, Bergen, Norway; ^2^Department of Medicine, Haukeland University Hospital, Bergen, Norway; ^3^Department of Medical Science, Faculty of Medicine, University of Bergen, Bergen, Norway; ^4^Department of Pathology, Haukeland University Hospital, Bergen, Norway

**Keywords:** HLH, cytomegalovirus (CMV), inflammatory bowel diseases (IBD), cytokines, immunosuppression

## Abstract

**Background:**

Hemophagocytic lymphohistiocytosis (HLH) is an immune mediated life-threatening condition. It is driven by an overactivation of the immune system and causes inflammatory tissue damage potentially leading to organ failure and death. Primary HLH is caused by genetic mutations, while secondary HLH is triggered by external factors. Viral infections are a well-known cause of secondary HLH. Cytomegalovirus (CMV) is a virus in the herpes family known to cause HLH in rare cases.

**Methods:**

We report a recent case of CMV-induced HLH, followed by a systematic review of described cases of this rare disease entity, through a structured search in the medical database PubMed. All articles were assessed on a predetermined set of inclusion criteria.

**Results:**

A total of 74 patients (age > 18 years) with CMV-related HLH were identified, 29 men, 42 women, and three patients with unspecified gender. Median age was 37.5 years (range 18–80). Sixty-six patients (88%) had one or more comorbid conditions and 22 patients (30%) had inflammatory bowel disease (IBD), the most frequent comorbidity. Forty patients (54%) received some form of immunomodulating treatment prior to HLH development. The general treatment approach was in general dual, consisting of antiviral treatment and specific immunomodulating HLH treatment approaches. Treatment outcome was at 77% survival, while 23% had fatal outcome.

**Conclusion:**

The findings highlight the importance of early diagnostic work up and treatment intervention. Ability to recognize the characteristic clinical traits and perform specific HLH diagnostic workup are key factors to ensure targeted diagnostic work and treatment intervention for this patient group.

## Introduction

Hemophagocytic lymphohistiocytosis (HLH) is a rare, immune mediated disease, characterized by lack of immune modulation, leading to uncontrolled activation of T-cells and macrophages, systemic inflammation, and tissue damage. The potential consequences are endothelial and tissue damage, thereby potentially causing multi organ failure and is associated with high morbidity and mortality rates. HLH can roughly be divided in two subgroups: primary and secondary HLH. Primary, or familial, HLH debuts at an early age, often within the first year of life (1). It is associated with genetic mutations causing inadequate functioning of the regulatory components of the immune system. Secondary, or acquired HLH, is caused by external influence or triggers. Some known triggers are infection, malignancy, autoimmune disease, immunosuppression, and organ transplantation (2–4). The pathophysiology for primary and secondary HLH are similar, causing immune hyperactivation followed by cytokine storm and inappropriate macrophage activation. The HLH diagnosis is defined by the present of five or more of the eight diagnostic HLH-2004 criteria (2, 5). The criteria include fever, cytopenia, splenomegaly, hyperferritinemia, hypertriglyceridemia/hypofibrinogenemia, histopathological hemophagocytosis, increased soluble CD25/IL-2 receptor levels (IL-2R), and reduced/absent natural killer (NK)-cell activity. The sensitivity and specificity for IL-2R are reported to be excellent, although supporting data have been scarce and needs validation (6). NK-cell activity and cytotoxicity assays are difficult to standardize, but will normally provide sufficient sensitivity and specificity (7). However, Perforin and CD107a have been found to be superior to NK-cell activity in diagnosing genetic HLH, and it has been advocated to include these test in the diagnostics (8).

The treatment principles of HLH aims to eliminate the underlying cause, if any, and to reduce the ongoing hyperinflammation. Treatment of any underlying cause depends on the identification and elimination of possible triggers, while hyperinflammation is primarily treated through immunomodulation (9). We report a recent case of cytomegalovirus (CMV)-induced HLH in an immunocompromised patient. Further, we performed a systematic review regarding published cases of concomitant CMV diseases and HLH. We focus on clinical manifestation, diagnostic examinations, clinical and laboratory findings, treatment approaches, and outcomes. We aim to provide a well-structured summary of the key aspects of the clinical features and treatment of the rare clinical cases of CMV-induced HLH.

## Case Presentation

A 23 old female, was previously diagnosed with ulcerous colitis and was treated with azathioprine 50 mg × 3 for 1 year. She had a history of iron deficiency anemia secondary to her inflammatory bowel disease (IBD) which needed treatment with iron infusions. Otherwise, the patient was previously healthy.

The patient's initial symptoms were dyspnea and a slight cough, as well as fever and general feeling of malaise. Over the next weeks, her condition progressed as she developed a dry cough, reduced appetite due to reduced sense of smell, and night sweats. The patient sought medical attention after a symptomatic period of 14 days. Upon arrival at the primary hospital, she presented with a dry cough, malaise, and night sweat. She had tachycardia with a pulse of 123 bpm. General blood tests were evaluated and are presented in Table 1. The patient had pancytopenia affecting all three cell lines, elevated liver enzymes, and a strikingly high ferritin (Table 1). Based on her respiratory symptoms and elevated D-dimer, a pulmonal computed tomography (CT) was taken on suspicion of pulmonary embolism, and the diagnosis was confirmed by an embolus in the left lower lobe. Treatment with low weight molecular heparin (LWMH) was initiated, and azathioprine was discontinued.

**Table 1:** Initial blood tests of the patient upon admission.

**Parameter**	**Results**	**Reference range**
Hemoglobin (g/dL)	11.1	11.7–15.3
Thrombocytes (× 10^9^/L)	62	165–387
Leukocytes (× 10^9^/L)	1.1	3.5–10
Ferritin (μg/L)	27,080	15–200
D-dimer (mg/L FEU)	>4	<0.50
ALAT (U/L)	50	10–45
GT (U/L)	72	10–45
LDH (U/L)	784	105–205

**Figure 1:**
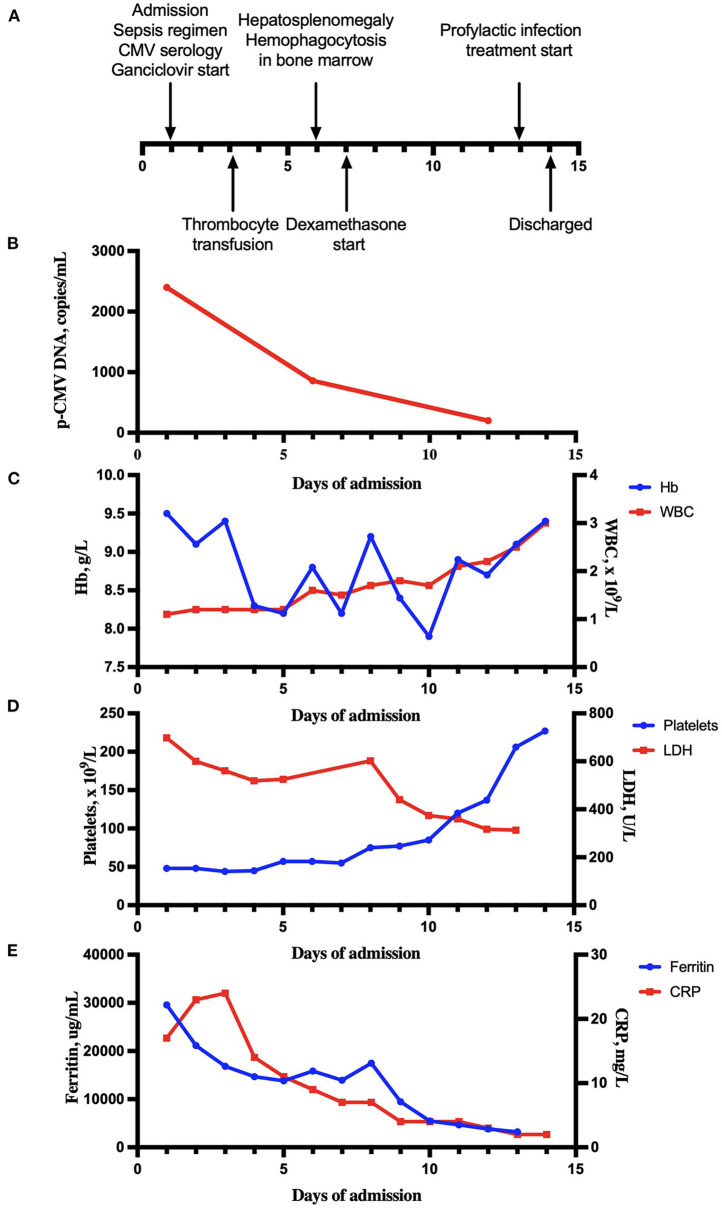
Clinical and laboratory evaluation during hospitalization. **(A)** A timeline of diagnostic and therapeutic events over the course of admission. **(B)** Plasma CMV-DNA PCR transcript levels over the course of treatment. **(C)** Hemoglobin (Hb) and white blood cell count (WBC) during hospitalization. **(D)** Platelets and lactate dehydrogenase (LDH) during hospitalization. **(E)** Ferritin and C-reactive protein (CRP) during hospitalization.

Based on the symptoms and initial blood test, investigations for possible hematological conditions were initiated, and the patient was transferred to a university hospital the following day. Her vitals upon arrival demonstrated blood pressure of 104/58 mmHg, tachycardia of 134 bpm, tachypnoea at 22/min, and a fever of 40.0°C. Based on suspicion of sepsis of unknown origin, a combination regimen of penicillin 3 g × 4 and gentamycin 350 mg × 1 was initiated. Both infections of the airways and the urinary tract were considered as possible causes. A thorough viral screening was performed to detect any possible infection as the cause of her neutropenic fever and airway symptoms. The results indicated previously undergone Epstein-Barr virus (EBV) infection, and an active or recent CMV infection due to high CMV immunoglobulin (Ig)G and IgM. Polymerase chain reaction (PCR) for CMV-DNA in serum revealed 2,400 copies/mL, further confirming an active CMV infection. Figure 1A presents a timeline of diagnostic and treatment interventions. Antiviral treatment with ganciclovir intravenously for CMV-infection was initiated, and the response was evaluated by reduction in CMV copy numbers by PCR (Figure 1B).

**Figure 2:**
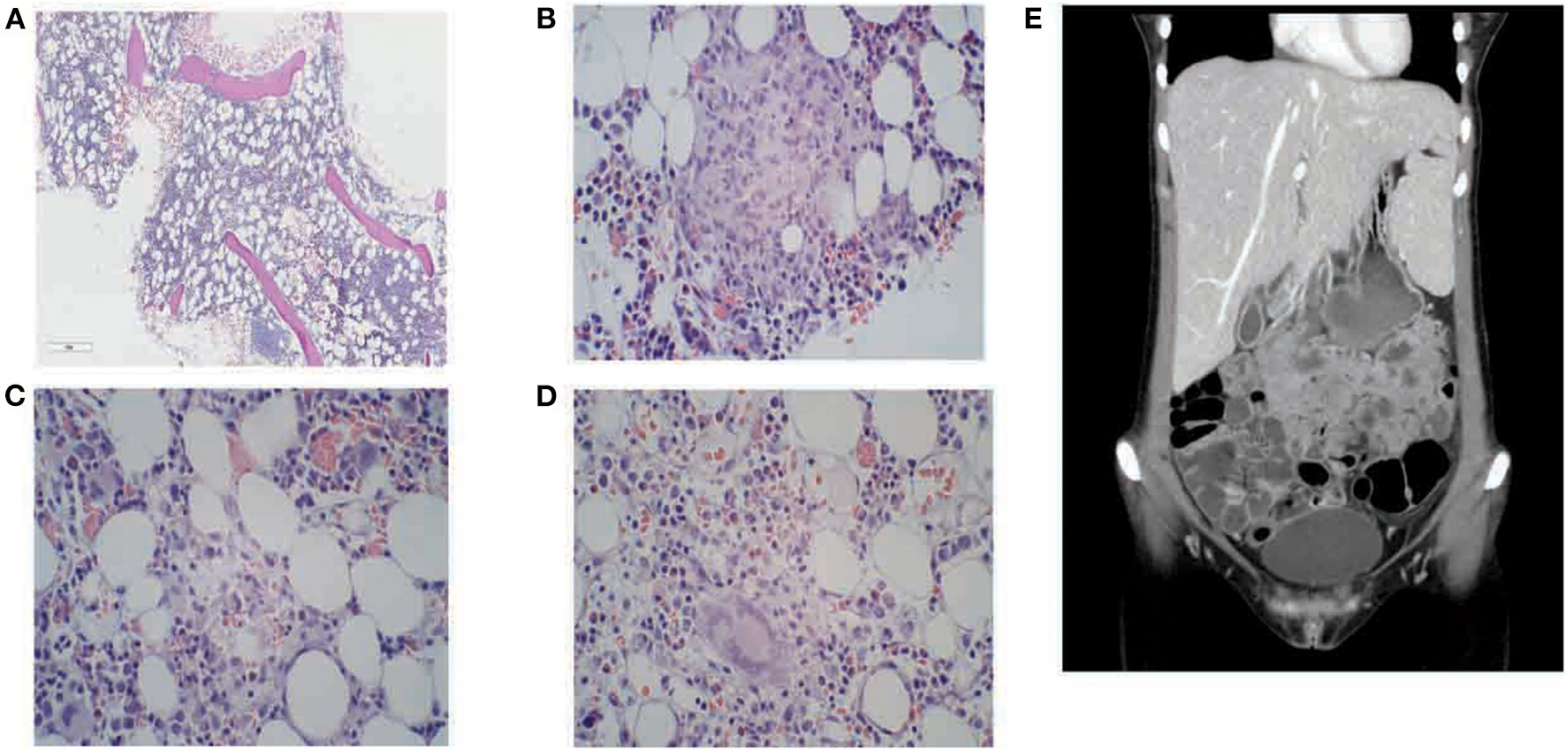
Bone marrow biopsy and CT examination. Bone marrow biopsy from the patient demonstrating; **(A)** modest hypocellularity; **(B)** higher magnification demonstrating granuloma formation with inflammation; **(C)** macrophages with marked hemophagocytosis; **(D)** giant cells. **(E)** CT abdomen showing hepatosplenomegaly with “kissing sign”.

Already the day after antiviral treatment was started, clinical improvement was seen. There was a slight rise in all three hematopoietic cell lines (Figures 1C,D). Ferritin serum levels declined from 29,587 to 21,130 μg/L (Figure 1E). On day 5, antiviral treatment was changed from intravenous ganciclovir to oral valganciclovir, 900 mg × 2. LMWH doses had been increased up to full dose, divided into two doses a day. The patient received thrombocyte transfusions to keep platelet levels > 50 × 10^9^/L. Bone marrow failure caused by either hematological malignancy or as a side effect of azathioprine treatment, was considered the most likely explanation for her pancytopenia. However, based on characteristic symptoms with fever, cytopenia and significantly high ferritin, the suspicion of HLH was raised. Further diagnostic work was targeted toward confirming a HLH diagnosis. A CT scan of the neck, abdomen, and pelvis was done to reveal any enlarged lymph nodes or organomegaly of the liver or spleen. Bone marrow aspiration and biopsy were performed on the first day of hospitalization. The results of the bone marrow examinations showed hypocellularity, granulomatous inflammation, and other reactive changes associated with HLH (Figures 2A–D). Hemophagocytosis was verified. As for the CT, no lymph nodes of pathological size were found, although severe hepatosplenomegaly with so-called “kissing sign” was present (Figure 2E), a radiological phenomenon seen in cases of severe hepatosplenomegaly (10). At this point, the patient fulfilled six of the total eight diagnostic criteria for HLH; fever, hyperferritinemia, pancytopenia, hypertriglyceridemia, splenomegaly, and verified hemophagocytosis in the bone marrow. An overview of the fulfilled criteria is seen in Table 2. There was enough clinical evidence to establish a HLH diagnose. She also had liver affection with hepatosplenomegaly and elevated liver enzymes, which further strengthened the diagnosis. For further clarification of the condition, tests for soluble IL-2R and NK-cell activity were requestioned. With an established clinical diagnosis of HLH, specific targeted HLH-treatment could be initiated. Treatment protocol was based on CMV load, and on an estimate of the patients' overall risk level. Initial treatment was a combination of continued antiviral therapy, and HLH-specific immunomodulation treatment with dexamethasone. A treatment plan with dexamethasone for a total duration of seven weeks was started on day 7 of hospitalization. Initial dose of dexamethasone was 16 mg, with a continuous tapering over the following weeks. The patient responded well to treatment with valganciclovir, as the viral count had lowered from the initial 2,400 to 859 copies/mL. Valganciclovir was continued for a maximum period of 21 days.

**Table 2:** HLH criteria met in the patient.

**Criteria**	**Presence**	**Highest/lowest value**	**Reference range**
Fever	Yes		
Cytopenias	Yes	Hb 7.9 g/dL	11.7–15.3
		Thrombocytes 48 × 10^9^/L	165–387
		WBC 0.9 × 10^9^/L	3.5–10
Splenomegaly	Yes		
Hypertriglyceridemia and/or hypofibrinogenemia	Yes	Triglycerides 318 mg/dL	<260 mg/dL
		Fibrinogen 2.8 g/L	1.9–4.0 g/L
Hyperferritinemia	Yes	29,587 μg/L	15–200 μg/L
Hemophagocytosis in bone marrow	Yes		
Elevated s-IL2 R	Yes	3,209 IU/L	160–620 IU/mL
Reduced/absent NK-cell activity	No		

*The patient met a total of seven out of eight criteria during the course of disease*.

Treatment with etoposide in addition to dexamethasone is considered indicated in severe cases of HLH. The need for etoposide in this patient was assessed by evaluating of her overall risk. Clinical condition, hematological and biochemical parameters, and soluble IL-2R levels were all used to assess the severity of her condition. Based on these considerations, her condition was classified as lower risk HLH. Furthermore, the chance that etoposide could re-trigger and potentially worsen the underlying CMV infection was also considered, and hence etoposide treatment was not initiated. Increased levels of soluble IL-2R also meant that the patient now fulfilled seven diagnostic criteria, lacking only reduced NK-cell activity. The results of the test for reduced NK-cell activity were inconclusive. Over the next days, the patient continued to respond well to treatment. Her ferritin levels decreased further, and her overall clinical state improved. A rise in all three cell lines was observed (Figures 1C,D).

The patient was discharged 14 days after admission. There was a clear clinical improvement from treatment. Her CMV count had dropped <150 copies/L, and her hematological parameters were normalizing. Upon discharge, all treatment was continued. She was at this point treated with dexamethasone and valganciclovir for CMV induced HLH, in addition to LWMH for pulmonary embolism.

## Materials and Methods

**Figure 3:**
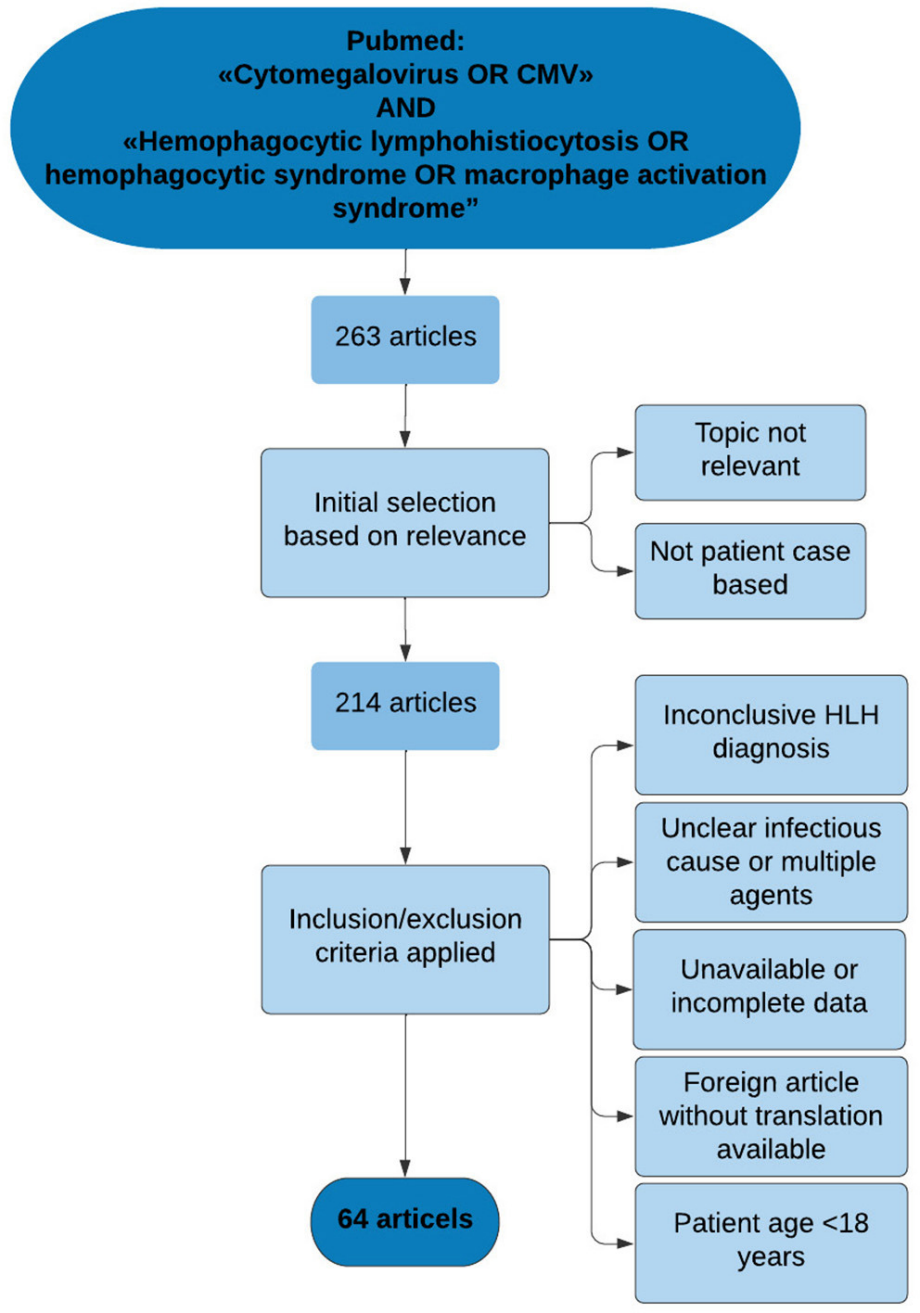
Flow chart of literature search process. The search and elimination process illustrated by a flow chart. The search led to 64 articles describing patient cases of CMV-associated HLH. Six of the articles described more than one relevant patient case. In total, 73 patient cases were included from literature.

Given the rarity of CMV induced HLH, and lack of clinical consensus, we performed a review of the literature regarding this disease. The aim of the literature review was to identify articles of clinical relevance in regards of clinical features, investigations and treatment presented in each case, emphasizing similarities in clinical presentation, diagnostic work and treatment regimens. A structured search of relevant literature was performed in the online database PubMed by January 2021 and reviewed by January 2022. The search in PubMed was performed combining the following search terms; “Cytomegalovirus OR CMV” and “Hemophagocytic lymphohistiocytosis OR hemophagocytic syndrome” and “Macrophage activation syndrome” (Figure 3). The initial search led to a total of 263 identified articles. The articles identified in the initial search were reviewed for relevance in a primary round of inclusion based on the following criteria: articles unrelated to the topic, or articles not describing patient cases. The process led to a total of 214 articles.

Further, the remaining articles were reviewed for confirmed diagnosis of CMV-induced HLH based on the following criteria: (i) symptoms and findings in accordance with the HLH-2004 diagnostic criteria, (ii) positive CMV serology and/or positive PCR in serum and/or other body fluids, and no other suspected primary cause of the condition. Furthermore, the following of exclusion criteria was made for elimination of non-relevant articles; (i) known primary HLH, or positive genetic test for primary HLH, (ii) malignant condition unknown prior to HLH development or discovered during investigation, (iii) or signs of malignancy discovered during examinations of the bone marrow, (iv) active co-infection with other HLH-triggering pathogens, for example EBV or herpes simplex virus (HSV), where both pathogens were considered equally possible causes of disease, (v) unclear or non-confirmed cause of HLH, (vi) articles not written in English, where no translation was available, (vii) patient age <18 years. An age cutoff was set to 18 years to exclude pediatric cases which have the possibility of an underlying genetic cause of the disease.

The result was 64 articles meeting the predetermined criteria for inclusion. Six of the articles described more than one patient case. Additionally, the case reported in this study was included in the overall results resulting in 74 identified cases. Figure 3 illustrates the selection process as a flow chart.

After selection, all clinical data of interest was extracted. Symptoms, findings, investigation methods, treatment, and outcome were the main data of interest. The information obtained was further used to present a structured overview of the clinical cases, approaches, and outcomes.

The results are present as a systematic review of the literature on CMV-associated HLH.

## Results

### Epidemiology

**Figure 4:**
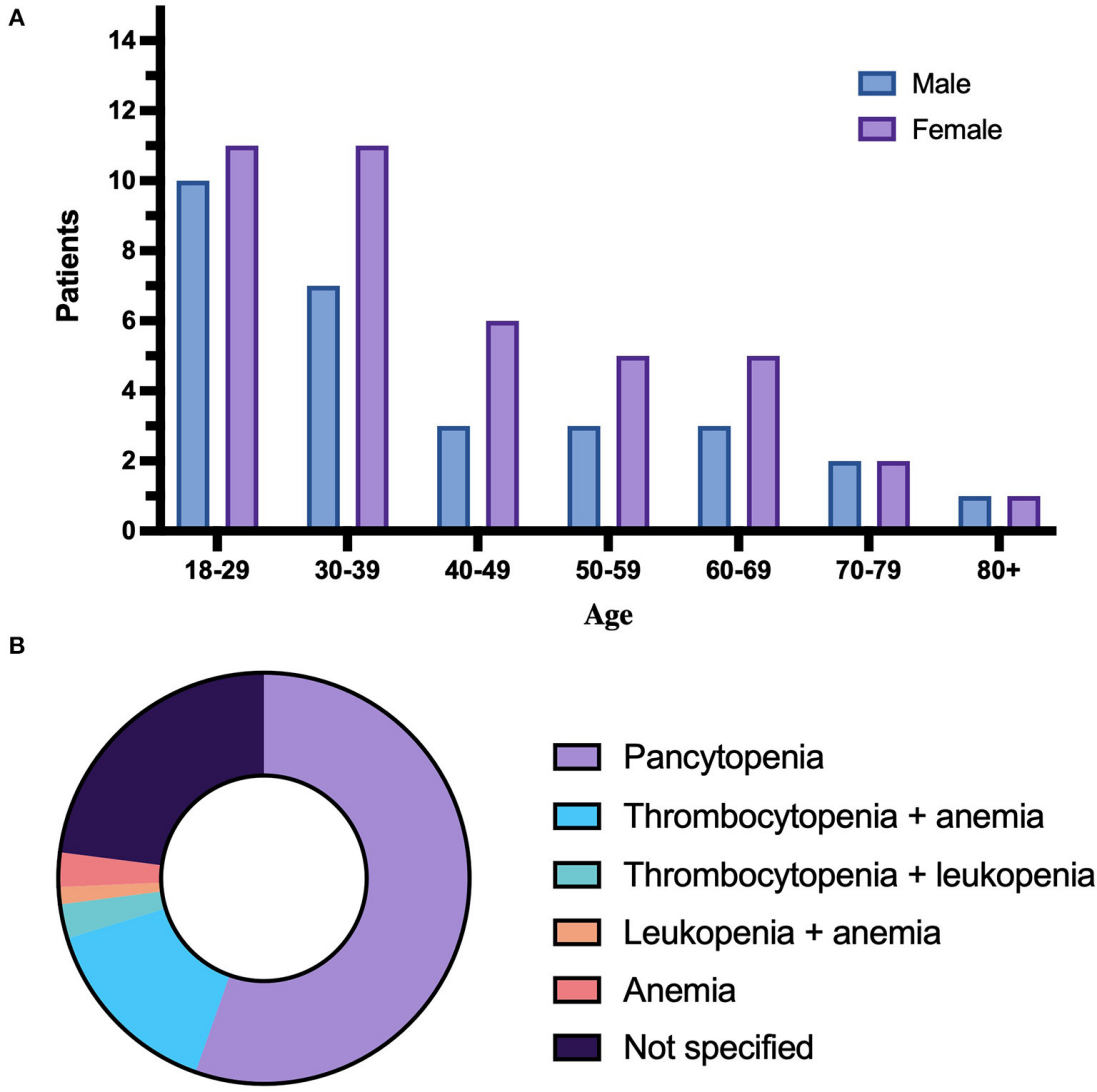
Distribution of age, gender, and cytopenias. **(A)** Illustrates distribution of age and gender. Patients of unknown age/gender are not included. **(B)** Illustrates the distribution of degrees of cytopenia.

The patient group consisted of 29 men and 42 women. Three patients were of unspecified gender (Table 3). The age ranged from 18 to 80 years, with a median age of 37.5 years (Table 3). Figure 4A illustrates distribution of age and gender in the patient group.

**Table 3:** An overview of the clinical findings upon arrival/time of diagnosis described in literature, including confirmed diagnostic findings among the number of patients investigated for each parameter.

	**Median**	**Range**	**Number (percentage)**	**Reference range** **M/F)**
**Sex**				
Male			29/74 (39%)	
Female			42/74 (57%)	
Unspecified			3/74 (4%)	
**Age**, years	37.5	18–80		
**Fever** (°C)	39.4	38–41.2	71/71 (100%)	
**Hepatosplenomegaly**			39/52 (75%)	
(liver and/or spleen)				
**Hemoglobin** (g/dL)	9.4	4.6–13.9	27/47 (57%)	13.4–17.0/11.7–15.3
**Thrombocytes** (× 10^9^/L)	72	4.9–305	43/51 (84%)	145–348/165–387
**WBC** (× 10^9^/L)	2.6	0.4–20	35/46 (76%)	3.5–10
**Ferritin** (μg/L)	6,052	587–86,000	61/62 (98%)	20–300/15–200
**Triglycerides** (mg/dL)	287	85–1,500	27/40 (68%)	<260
**Fibrinogen** (g/L)	1.4	0.3–2.8	11/16 (69%)	1.9–4.0
**Hemophagocytosis** (bone marrow)			62/67 (93%)	
**Soluble IL-2R** (U/mL)	3,606	212–25,568	13/18 (72%)	160–620
**NK-cell activity**			6/8 (75%)	

### Clinical Presentation and Findings

An overview of all significant clinical findings in the patient group are summarized in Table 3. Fever was main recurring symptom in the patient group. Seventy-one patients presented with fever at some point during the course of disease. Temperature was specified in 29 patients. The described temperatures ranged from 38.0 to 41.2°C, with a median temperature of 39.4°C.

Organomegaly with enlargement of either the liver, the spleen, or both was described in 39 patients. Three patients had hepatomegaly only, and 18 had splenomegaly only, while enlargement of both spleen and liver was found in 18 patients. Thirteen patients were investigated but had neither, and presence of hepatosplenomegaly was unspecified in 22 patients.

Cytopenia as a diagnostic criterion in HLH patients is defined as low cell count in two or more blood cell lines. Pancytopenia was described in 41 patients, 14 patients had cytopenia in two lines. Eleven of the 14 patients had low platelets and anemia, with high or normal white blood cell count (WBC), and two patients had low platelets and leukopenia, with a normal red blood cell count. One patient had anemia and leukopenia, with normal platelet count. Two patients did not qualify for cytopenia per diagnostic definition. In the remaining 17 patients, the cytopenia was unspecified or the values used were not translatable to the standard unit used for comparing data. Two of the patients with unspecified cytopenia were confirmed to have values within the diagnostic range. An overview of the distribution of cytopenia in the patient group is presented in Figure 4B. Anemia in the patient group was described in two ways: As low Hb levels, or as low hematocrit in percent. Hb levels were specified in 47 patients, and below normal range in 44 patients. Further, levels < 9 g/dL were present in 27 patients at some point during the course of disease. Hematocrit was specified in five patients, and four patients were described as anemic by low hematocrit value. Thrombocyte levels were described in 51 patients. Thrombocytopenia below the diagnostic cutoff of <100 × 10^9^/L was present at some point in 43 patients. White blood cell count was specified in 46 patients. Leukopenia, defined as WBC < 4.0 × 10^9^/L, occurred in 35 patients. Leukocytosis, defined as WBC >10 × 10^9^/L in adults, occurred at some point during the course of disease in eight patients.

Hyperferritinemia above the HLH diagnostic limit of 500 μg/was described in 61 patients. Fifty-six of these cases had specified values. Ferritin values in the patient group ranged from 587 to 82,000 μg/L, with a median value of 6,051.5 μg/L. Hypertriglyceridemia above 265 mg/dL and/or hypofibrinogeneima below 1.5 g/L was present in 32 patients. Six of the patients had both hypertriglyceridemia *and* hypofibrinogeneima. Hypertriglyceridemia alone was described in 21 cases. Hypofibrinogeneima alone was present in five cases. Ten patients had values within normal range, and 32 cases were unspecified. The triglyceride values ranged from 84 to 1,500 mg/dL, with a median value of 286.5 mg/dL. The fibrinogen values ranged from 0.3 to 2.8 g/L, with a median value of 1.4 g/L.

Soluble IL2 receptor levels were reported in 18 patients. IL-2R levels above 2,400 U/mL are associated with HLH. Thirteen of the 18 patients with increased IL-2R had values above the diagnostic cutoff. Elevated soluble IL-2R levels without further specification were reported in two patients. Reduced or absent NK-activity was present in six cases. Three patients were described to have reduced/decreased activity. NK activity was described as absent in the remaining three patients. In one patient, the NK cell activity was examined and described as normal. In one patient, the results were inconclusive due to insufficient test material. Bone marrow examinations are done by performing aspirations and/or biopsies of the bone marrow. HLH associated findings include hemophagocytosis and histiocytosis. Bone marrow examinations were performed in 67 cases. Within this group, hemophagocytosis was observed in 62 of the patients. Histiocytosis, including the description of macrophage activation and/or hyperplasia, was present in 42 patients. In two patients, hemophagocytosis was not found upon investigation, and in five patients, the presence of hemophagocytosis was not specified. In three of the unspecified cases, the presence of activated macrophages is described. Finally, in three cases, no bone marrow examinations were performed, and in two cases, hemophagocytosis was detected in other organs, the liver, and spleen.

### Comorbidity

Eight patients had no specified comorbidity or underlying condition, while the remaining 66 patients had some form of underlaying disease. Fourteen of these patients had more than one condition. Twenty-two patients suffered from IBD; 14 patients had Crohn's disease (CD) and eight had UC. Four patients were HIV-positive, and one of these had developed AIDS. Autoimmune disease was present in 10 patients; four had systemic lupus erythematosus (SLE), two had myasthenia gravis (MG), two had myositis, one had Sjogren's syndrome, and one had granulomatosis with polyangiitis (GPA). Eight patients had a previously known malignant conditions and included three patients with acute lymphoblastic leukemia (ALL), one with acute myeloid leukemia (AML), one with chronic lymphocytic leukemia (CLL), and three with other forms of malignant conditions. The patient group included a total of 15 transplant recipients. Five patients had undergone hematopoietic stem cell transplantation (HSCT); three allogeneic and two autologous transplantations, nine patients had undergone kidney transplantation, and one patient had undergone liver transplantation. Lifestyle-associated conditions such as diabetes type II, hypertension, and hyperlipidemia were present in eight patients. For two female patients, diagnosis was made during pregnancy (Table 4).

**Table 4:** Comorbidity present in the patient group.

**Comorbidity**	**Number**	**Percentage**
**No comorbidity**	**8**	**10.8%**
**IBD**	**22**	**29.7%**
Mb Crohn	14	18.9%
Ulcerous colitis	8	10.8%
**Autoimmune disease**	**10**	**13.5%**
SLE	4	5.4%
Myasthenia gravis	2	2.7%
Sjogren's syndrome	1	1.4%
Myositis (inflammatory-/dermatomyositis)	2	2.7%
Granulomatosis with polyangiitis	1	1.4%
**Malignancy**	**8**	**10.8%**
AML	1	1.4%
ALL	3	4.1%
CLL	1	1.4%
Other	3	4.1%
**Infection**	**7**	**9.5%**
HIV/AIDS	5	6.8%
Endocarditis	1	1.4%
Tuberculosis	1	1.4%
**Transplant recipients**	**15**	**20.3%**
Kidney	9	12.2%
Liver	1	1.4%
Stem cell transplant	5	6.8%
Allogeneic	3	4.1%
Autologous	2	2.7%
**Lifestyle associated conditions**	**8**	**10.8%**
Obesity	2	2.7%
Diabetes type II	2	2.7%
Hypertension	3	4.1%
Hyperlipidemia	1	1.4%
**Pregnancy**	2	2.7%
**Other** (IBS, GATA mutation, AOSD, Bipolar	8	10.8%
disorder, LETM, and heart failure)		

*IBD, inflammatory bowel syndrome; SLE, systemic lupus erythematosus; AML, acute myeloid leukemia; ALL, acute lymphoblastic leukemia; CLL, chronic lymphocytic leukemia; HIV, human immunodeficiency virus; AIDS, acute immunodeficiency syndrome; IBS, irritable bowel syndrome; AOSD, adult-onset Still's disease; LETM, longitudinal extensive transverse myelitis*.

### Diagnostic Clinical Criteria

The clinical criteria for HLH include eight symptoms and finding associated with HLH. The presence of five or more criteria are considered diagnostic for HLH. Fever was the most frequently fulfilled criteria accounted for, present in 71 patients. Hemophagocytosis in the bone marrow was found in 62 patients. Hyperferritinemia > 500 μg/mL was present in 61 patients. Cytopenia within the diagnostic range in at least two cell lines was present in 57 patients. Thirty-six patients met the criterion for splenomegaly. Thirty-two patients had hypertriglyceridemia and/or hypofibrinogenemia. Increased soluble IL-2R > 2,400 IU was present in 13 patients. Reduced NK-cell activity was specified in six patients.

Out of the 74 patients in the group, 39 patients fulfilled five criteria or more, from which two patients met all eight criteria. Thirty-five patients met <5 criteria. The median number of criteria met was five, while the average number was 4.6, ranging from two to eight criteria. Table 5 provides a full overview of the fulfilled criteria in each patient in the group.

**Table 5:** Presence or absence of HLH-2004 clinical criteria in each patient reviewed.

**Patient**	**Sex**	**Age**	**Comorbidity**	**Fever**	**Cytopenia**	**Splenomegaly**	**Ferritin**	**Tiglycerides/fibrinogen**	**Reduced NK-cell activity**	**s-IL2 R**	**Hemophagocytosis**	**SUM**	**References**
1	M	18										6	(11)
2	M	71										4	(12)
3	M	27	HIV									5	(13)
4	M	63	Mb Crohn									4	(14)
5	M	35	Mb Crohn									5	(15)
6	F	52	UC									5	(16)
7	F	32	UC									4	(17)
8	M	22	Mb Crohn									3	(17)
9	F	32	Mb Crohn									6	(18)
10	F	28	Mb Crohn									4	(19)
11	F	33	Mb Crohn									4	(19)
12	F	30	Mb Crohn									3	(19)
13	F	38	Mb Crohn									4	(19)
14	M	36	Mb Crohn									4	(20)
15	M	21	HIV									7	(21)
16	F	70	Sjogren, HT									6	(22)
17	M	72	HT, HL									3	(23)
18	M	21										5	(24)
19	F	32										6	(25)
20	F	48	IBS									5	(26)
21	F	39	Obesity, DIA									8	(27)
22	X	30	HSCT									2	(28)
23	X	18	HSCT									2	(28)
24	X	56	HSCT									2	(28)
25	F	50	KTx									4	(29)
26	F	22	SLE									3	(30)
27	F	37	SLE									6	(31)
28	M	35	UC									6	(32)
29	F	20	Mb Crohn									5	(33)
30	M	22	Endocarditis									3	(34)
31	F	44	SLE									6	(35)
32	M	29	HIV									5	(36)
33	F	40	PL, HSCT									6	(37)
34	M	62	CLL									5	(38)
35	F	52	AML, HSCT									2	(39)
36	F	27										6	(40)
37	F	35	Pregnant									5	(41)
38	F	21	IM, obesity									7	(42)
39	M	25	SLE									4	(43)
40	M	63	KTx, DIA, HT									3	(44)
41	F	24	Mb Crohn									4	(45)
42	M	40	KTx									5	(46)
43	F	39	Pregnant, KTx									6	(47)
44	F	22	GATA mutation									7	(48)
45	F	31	MG									5	(49)
46	M	22	Mb Crohn									3	(50)
47	M	34	AIDS, BL									3	(51)
48	F	69										4	(52)
49	F	44	UC									5	(53)
50	F	59	UC									2	(54)
51	F	63	KTx, DIA									6	(55)
52	F	47	MG									8	(56)
53	F	80	AOSD									3	(57)
54	M	51										6	(58)
55	M	X	UC, BPD									5	(59)
56	F	18	ALL									5	(60)
57	F	X	DM									5	(61)
58	M	38	ALL									4	(62)
59	F	49	LTx									4	(63)
60	F	26	AOSD									1	(64)
61	M	80	Brain tumor, TB									6	(65)
62	M	48	LETM									5	(66)
63	M	50	KTx									3	(67)
64	M	35	KTx									4	(67)
65	M	50	UC, HIV									5	(68)
66	F	51	Heart failure									6	(69)
67	F	69	GPA									4	(70)
68	F	63	Mb Crohn									6	(71)
69	M	23	Mb Crohn									4	(71)
70	F	62	ALL									5	(72)
71	M	46	KTx									3	(73)
72	F	28	KTx									3	(73)
73	F	72										2	(74)
74	F	23	UC									7	Present
SUM				71	57	36	61	32	6	13	62		

*The table illustrates which of the diagnostic criteria are present in each patient. Red cells illustrate that a criterion is confirmed present, green cells mean that a criterion is confirmed not present, and white cells represent data unspecified*.

*X, Age or gender unknown/unspecified*.

*HIV, human immunodeficiency virus; UC, ulcerous colitis; HT, hypertension; HL, hyperlipidemia; IBS, irritable bowel syndrome; DIA, diabetes; HSCT, hematopoietic stem cell transplantation; KTx, kidney transplantation; SLE, systemic lupus erythematosus; PL, promyelocytic leukemia; CLL, chronic lymphatic leukemia; AML, acute myeloid leukemia; IM, inflammatory myositis; MG, myasthenia gravis; AIDS, acute immunodeficiency syndrome; BL, Burkitt lymphoma; AOSD, adult-onset Still's disease; BPD, bipolar disorder; DM, dermatomyositis; LTx, liver transplantation; TB, tuberculosis; LETM, longitudinal extensive transverse myelitis; GPA, granulomatosis with polyangiitis*.

### Viral Serology

To identify the underlying cause of disease, the diagnostic tools used were viral serology and PCR of peripheral blood, serum, urine, or other body fluids. Thirty-seven patients were described to have positive CMV serology. In 45 cases, PCR was used for detection of CMV-DNA and to determine the viral load.

### Treatment and Outcome

All articles included in this review describe HLH associated with CMV disease. Therefore, the general treatment principle for the patient group consisted of a combination of antiviral treatment and targeted, anti-inflammatory HLH treatment. Sixty-seven patients received some form of antiviral treatment. Ganciclovir was used in 59 of the cases. Forty-one patients received ganciclovir as the only antiviral treatment, while 18 patients received additional treatment with Valganciclovir or Foscarnet. Four patients received valganciclovir as their only antiviral treatment. Foscarnet was given in addition to ganciclovir and/or valganciclovir in six cases. Ten patients received no antiviral treatment at all.

The HLH targeted treatment described in the literature reviewed consisted of different immunomodulating regimens. Fifty-eight patients were treated with some form of corticosteroids. Twenty-nine of these received corticosteroids as their only immunomodulating treatment. Cytostatic therapy with etoposide was used in addition to immunomodulating treatment in 10 cases. Besides corticosteroids, some other forms of immunosuppressive treatments were also used. Intravenous immunoglobulin (IVIG) was used in a total of 26 cases. Ten patients were treated with cyclosporine A, and two patients received tumor necrosis factor-α (TNF-α) inhibitors. For 10 patients, no immunomodulating treatment is described. For two patient cases, no treatment protocol was specified at all (Table 6).

**Table 6:** The distribution of use of each treatment option in the patient group.

**Treatment**		**Number**	**Percent**
Antiviral	Ganciclovir	59	79.7%
	Valganciclovir	21	28.4%
	Foscarnet	6	8.1%
Cytostatic	Etoposide	10	13.5%
Immunomodulating	Corticosteroids	58	78.4%
	IVIG	26	35.1%
	Cyclosporine A	10	13.5%
	Anti-TNF-α	2	2.7%
	G-CSF	5	6.8%
Support treatment	Blood transfusion	7	9.5%
	Antibiotics	27	36.5%
Other		6	8.1%

*Many patients received more than one type of treatment, and more than one drug withing the same treatment group*.

*IVIG, intravenous immunoglobulin; G-SCF, granulocyte stimulating colony factor; anti-TNF-α, anti-tumor*.

Supportive treatment was provided as required. Seven patients required blood transfusion as part of their treatment. Twenty-seven patients received antibiotics at some point during treatment, and 15 of these received antibiotics as initial treatment, before a HLH diagnosis was set.

In all cases in which patients used immunomodulating treatment prior to HLH development, the treatment was discontinued, this affected 40 patients.

From the 74 patients included in this study, 57 patients responded well to treatment and survived, while 17 patients died. The survival rate in the patient group was 77%. The main causes of death, where specified, were multi organ failure and respiratory failure.

## Discussion

Infection is known to be one of the most common causes of secondary HLH. When it comes to virus associated HLH, EBV is the most common agent. However, HLH could also be associated with other viruses in the herpesvirus family, including CMV (1, 75). CMV infection is common, with a high prevalence of seropositivity varying with age and geographic location (76, 77). Transmission often takes place early in life, and infection with CMV often has an asymptomatic or subclinical presentation in immunocompetent patients. Some factors are associated with higher risk of more severe infection and infection-related complications, including HLH development (78, 79).

In the present study we identified cases with CMV associated HLH, and found a heterogenous patient group, with a great variety in age and comorbidity. Pediatric patients under the age of 18 were excluded. The overall gender distribution had a slightly higher percentage of female patients. The younger patient group, from 18 to 29 years old, had an equal distribution of male and female patients, while the majority of the middle-aged patients, between 30 and 59 years, were female. IBD was the most common comorbidity observed, and these patients were often treated with immunomodulation such as azathioprine or mesalazine. The results suggest that IBD is the most frequently occurring risk factors in CMV-associated HLH patients described in literature, as also present in our case. The correlation between IBD and HLH development is a well-known topic in literature. Bambrilla et al. presented a systematic review regarding patients with IBD and infectious HLH. They found an association between the use om immunomodulating treatment for IBD, and HLH development (80). The patient group reviewed presented with the same symptoms as described in the present study. Other frequently reported underlying causes were organ or HSCT, and autoimmune diseases. The use of immunomodulating treatment before HLH development was frequently recurring, concerning 54% of the patients. This correlated well with the most common comorbid conditions in the patient group; IBD, autoimmune disease, and organ transplantation, or HSCT are all conditions requiring immunosuppressive treatment. Although there was a high number of immunocompromised patients, CMV-triggered HLH was also seen in otherwise healthy patients as well, as eight (12%) of the identified cases were previously healthy patients. This illustrates that though it is possible to develop HLH due to CMV infection in healthy individuals, the risk is significantly higher in the presence of immune-related comorbidity and immunosuppressive treatment. Conclusions on incidence should however be avoided in case series as a number of factors will bias publicized cases.

The initial symptoms of HLH can be vague and unspecific. Fever and malaise were two frequently reported symptoms in the patients upon arrival. These symptoms can easily be mistaken for a non-systemic infection or even sepsis. A significant number of patients received initial treatment with antibiotics on suspicion of an ongoing bacterial infection. A common trait in these patients were a progressive worsening of their condition despite the attempted treatment. Characteristic initial findings were fever and hyperferritinemia. Ferritin reached strikingly high levels, with values reaching 40–50,000 ug/mL in some cases. Unlike fever, which was a highly frequent, although unspecific finding, hyperferritinemia raised suspicion of an inflammatory or hematological condition. Cytopenia was detected in initial blood tests and worsening of the cytopenia typical occurred during the course of disease. Therefore, cytopenia was a developing criterion, and some patients met the criterion during course of disease instead of upon admission. Pop et al. (14) presented a patient blood values within normal range upon arrival, although with rapidly progression of pancytopenia, a similar clinical course seen in other patients (26, 43, 59). Although HLH is associated with cytopenia, leukocytosis occurred in several patients, likely a reactive increase due to inflammation.

Hemophagocytosis is primarily described in the bone marrow, although may also take place in the spleen, liver, or lymph nodes. In the identified cases of CMV associated HLH, hemophagocytosis was primarily identified in the bone marrow. However, hemophagocytosis in other organs was also described. Choi et al. (46) described hemophagocytosis in the liver of a patient, while Kohara et al. (50) described hemophagocytosis in the spleen. Although highly associated with the condition, hemophagocytosis is not pathognomonic for HLH. Hemophagocytosis may not be present at an early stage of disease, or HLH can be present without hemophagocytosis at all (3, 4). Normal findings in the bone marrow were observed in two patients reviewed in literature (26, 62).

The HLH-2004 criteria are based on the most frequently occurring clinical presentation of HLH patients, and include symptoms and findings described above. They reflect the cardinal signs of HLH and are useful for rapid recognition of the condition. Five of eight criteria are required to set a HLH diagnosis, however the number of criteria present in the patient group varied from one to all eight, reflecting an individual evaluation of each patients' clinical presentation. Furthermore, the results indicate that some findings are more emphasized than others in the diagnostic process. In the present study we used the term HLH based on the diagnosis made by treating physicians, although we cannot rule out that some patients would not meet all the diagnostic criteria for HLH, bases incomplete or lack of clinical information.

The variability in fulfillment of diagnostic criteria emphasize the broadness of clinical features and presentation within the HLH term. However, the diagnostic criteria can be very useful in the diagnostic process and set a clear frame for the condition and following treatment, and proper diagnostic workup should be performed in all patients with suspicion of HLH.

Rapid treatment of HLH is crucial to prevent progression of inflammatory damage, and to prevent mortality (3). The choice of treatment requires consideration of the triggering cause, grade of hyperinflammation, and overall clinical state of the patient. The standard protocol for HLH treatment is an 8-week regimen of corticosteroids and etoposide, with or without additional methotrexate (4, 81). In the literature reviewed, the general approaches were immunomodulation with a combination of corticosteroids, etoposide, and other immunomodulating drugs in accordance with the described protocol. Etoposide was used in treatment of 10 patients. The considerations for treatment with etoposide, were severity of disease and overall clinical state of the patient. The same considerations were made for the patient described in our present case report, and etoposide was not initiated based on the patient's clinical state and the risk of re-activation of the underlying infection. Some treatment options used in literature are at present time not included in the standard treatment protocol for HLH. Lau et al. (42) and Divithotawela et al. (53) described the use of anakinra, an interleukin-1 inhibitor, as a treatment option for CMV induced HLH. Furthermore, first-line treatment of CMV infection is ganciclovir or its prodrug valganciclovir (79). Ganciclovir was seen to be the superior viral treatment of choice for patients with CMV induced HLH. In addition to ganciclovir and valganciclovir, foscarnet was in some cases added to the antiviral treatment protocol. Ganciclovir is known to cause bone marrow suppression, which may be a reason itself to switch to foscarnet (82), on the counter side foscarnet will often pose a risk of kidney failure (83). In 26 cases the use of IVIG was initiated, although the documentation of IVIG in HLH treatment is somewhat limited. Jordan et al. (4) claims that IVIG has a place in treatment of patients with viral HLH, and Hot et al. (25) report successful treatment of a patient with virus associated HLH using IVIG only. Further studies should investigate the potential role of IVIG in HLH treatment.

With infectious HLH, there is a fine balance between immunomodulating treatment and infection control. During treatment, the patients may be exposed to two risks; re-activation of the original cause of disease or contracting a new infection. Janka and Lehmberg (1) further problematized this regarding general HLH treatment considerations, and treatment must reach a balance between sufficient immunosuppression, and infection control and protection. Fifty-four percent of the patients used immunomodulating treatment, such as azathioprine, mesalazine, or prednisolone, prior to HLH development, and immunomodulation puts the patients at risk both for infection itself and complications related to infection (77). In all these cases, the immunomodulating medication in question was discontinued. After HLH treatment, there are several considerations to be made regarding continued treatment of their comorbid condition, although precise data were limited.

Treatment response was monitored by clinical and biochemical parameters. Lowering fever, normalization of blood values such as cytopenia and hyperferritinemia, as well as improvement of overall general condition, indicated improvement. Ferritin and soluble IL-2R are known to reflect the disease activity well, making them suitable for monitoring treatment (4). The results depended on gathering, systemizing, and comparing clinical data from the literature reviewed. The literature showed many similarities in terms of pathophysiology, and the same protocols for diagnostics and treatment were referred to in most cases. Central clinical traits such as fever, cytopenia, and choice of treatment was overall well-described. This provided solid material for collecting and comparing data on the areas mentioned. However, there was also a significant inconstancy in report of other clinical features. Soluble IL-2R and NK-cell activity were very underreported in the literature, and many articles had no mentioning of either parameter. There was also great variety in available data on the symptomatic development during treatment, and this also include comparing data of laboratory parameters upon arrival and during/after treatment. A complete review of the literature on the areas where the available information was insufficient are hence a limitation of the study.

## Conclusion

We present a recent case of concomitant HLH and CMV infection in a young female patient with UC, and have performed an extensive literature review which identified 73 additional cases described with concomitant HLH and CMV infection. The age and gender distribution were heterogenous. A significant number of the patients had IBD, further strengthening the correlating between IBD, CMV infection, and HLH. The results also showed a recurrence of certain clinical findings and in treatment approaches. The survival rate in the group was high, indicating the importance of rapid and prompt diagnostic work with recognition of the cardinal signs of HLH. Treatment intervention following standard treatment protocols of antiviral treatment and immunomodulation are cornerstones in caring for these patients.

## Data Availability Statement

The original contributions presented in the study are included in the article/supplementary material, further inquiries can be directed to the corresponding author/s.

## Author Contributions

LR and HR: conceptualization, investigation, and writing—original draft preparation. LR, KM, LH, and HR: writing—review and editing. LR, LH, and HR: visualization. KM and HR: supervision. HR: project administration. All authors have read and agreed to the published version of the manuscript.

## Conflict of Interest

The authors declare that the research was conducted in the absence of any commercial or financial relationships that could be construed as a potential conflict of interest.

## Publisher's Note

All claims expressed in this article are solely those of the authors and do not necessarily represent those of their affiliated organizations, or those of the publisher, the editors and the reviewers. Any product that may be evaluated in this article, or claim that may be made by its manufacturer, is not guaranteed or endorsed by the publisher.
